# Semiological differences of functional seizures between pediatrics and adults: video electroencephalography analysis

**DOI:** 10.1186/s42494-025-00236-0

**Published:** 2026-02-02

**Authors:** Salsabil Abo Al-Azayem, Nirmeen A. Kishk, Rehab Magdy, Amani Nawito, Eman Hany Elsebaie, Mai Belal, Doaa Abdellatif Elelwany

**Affiliations:** 1 Department of Neurology, Faculty of Medicine, Cairo University, Cairo, 11956 Egypt; 2 Department of Clinical Neurophysiology, Faculty of Medicine, Cairo University, Cairo, 11956 Egypt; 3 Department of Public Health and Community Medicine, Faculty of Medicine, Cairo University, Cairo, 11956 Egypt; 4 Department of Psychiatry, Faculty of Medicine, Cairo University, Cairo, 11956 Egypt

**Keywords:** Functional seizures, Pediatric, Adults, Semiology, Video EEG

## Abstract

**Background:**

Diagnosing functional seizures can be challenging, and the semiology may vary between pediatric and adult age groups. Identifying those variabilities may be of diagnostic value. This study aimed to compare the semiological characteristics of functional seizures in both the pediatric and adult populations.

**Methods:**

All video ictal electroencephalogram (EEG) recordings at Cairo University Epilepsy Unit (CUEU) from January 2021 to December 2023 were retrospectively reviewed for adults or children with functional seizures. Detailed semiological characteristics of the ictal events were analyzed independently by at least two epileptologists. Each event was listed under the classification system as either major motor, minor motor, dialeptic, non-epileptic aura, or mixed type.

**Results:**

A total of 54 pediatric and 65 adult video ictal EEG studies were evaluated. Minor motor type was the most common clinical semiology among adult and pediatric groups, yet more prevalent in pediatric than adult patients (61.1% vs. 40.0%, *P* = 0.022). In comparison, the major motor events were significantly higher in adults than in pediatrics (25 event [38.5%] vs. 9 events [16.7%], *P* = 0.009). No statistically significant differences were found between pediatrics and adults regarding pelvic thrusting (3.7% vs. 9.2%), back arching (7.4% vs. 4.6%), clenched fists (9.3% vs. 15.4%), ictal pain (13.0% vs. 18.5%), ictal fear (5.6% vs. 3.1%), or ictal crying (7.4% vs. 4.6%), respectively.

**Conclusions:**

The semiology of functional seizures varies between pediatric and adult age groups; minor motor events predominate in pediatric patients, while the major motor type is more common in adults.

**Supplementary Information:**

The online version contains supplementary material available at 10.1186/s42494-025-00236-0.

## Background

Functional seizures are a subtype of functional neurological disorder (FND) characterized by paroxysmal episodes of altered awareness, movement, or sensation that resemble epileptic seizures but do not have accompanying epileptiform abnormalities on an EEG [1]. According to reports, it might take up to seven years to diagnose functional seizures [2]. Prior to receiving an accurate diagnosis, functional seizures may be misdiagnosed as epilepsy, subjecting patients to potentially harmful anti-seizure medications (ASMs) [3], benzodiazepine use during pseudostatus, prolonged school/work absences, and delay of receiving the proper psychological management [4].

In tertiary care epilepsy monitoring units (EMU), out of 20% to 40% of adults and 10% to 23% of children presenting with “drug-resistant epilepsy” were found to have functional seizures [5]. The incidence of functional seizures is 1.4–4.9 cases per 100,000 people per year and the prevalence range of 2–33/100,000 [4]. However, epilepsy can be associated with functional seizures with estimated comorbidity ranging from 5% to 50% [6].

Several works have studied clinical characteristics that aid in differentiating functional seizures from epileptic seizures [7–10]. However, functional seizures have been comprehensively described in the literature, mostly among adults and less frequently in pediatrics [11–13], which hinders its recognition and management in various age groups [14].

Understanding semiological differences in functional seizures between pediatrics and adults would aid in the early identification of functional seizures, optimizing the outcomes for children and adolescents with functional seizures. Furthermore, better interpretation of semiological variations can open the scope for future research on the different psychopathology backgrounds of functional seizures in special populations, ultimately improving management approaches. This is particularly relevant given the high heterogeneity in functional seizure management and the lack of clinical guidelines for children and adolescence [15, 16].

Therefore, this study aimed to determine whether the clinical characteristics of functional seizures vary between pediatric and adult populations, thus advancing our understanding of pediatric-onset functional seizures.

## Methods

### Study design and participants

Patients diagnosed with functional seizures based on video EEG monitoring in Cairo University Epilepsy Unit (CUEU) between January 2021 and December 2023 were retrospectively evaluated. At least two epileptologists independently reached an agreement on the diagnosis of functional seizures before commencing the research. As reported by Seneviratne et al. [11], they conducted a separate semiology analysis and independently filled up a predefined checklist containing detailed semiology analyses. A third researcher arbitrated cases of disagreement. The diagnosis of functional seizures relied on typical clinical semiology, with no concurrent abnormal ictal electrographic activity or postictal slowing observed during video-EEG monitoring.

Patients were categorized into two groups: a pediatric group (< 18 years) and an adult group (≥ 18 years).

Exclusion criteria included attributing these non-epileptic paroxysmal events to another diagnosis, such as parasomnias or movement disorders; failure to capture clear video-EEG that hampers an accurate semiological analysis of the event; and incomplete demographic data. The diagnosis of functional seizures in patients with pre-existing epilepsy is not considered an exclusion criterion.

### Data collection tools

Demographics and data about a family history of epilepsy or psychiatric illness were obtained. Regarding each documented clinical event, all included patients and their parents were inquired about the frequency of attacks/month, stereotypical pattern, either occurrence alone or in front of the public, and post-ictal manifestations.

Detailed visual analysis of the semiology of each clinical event was conducted independently by two epileptologists (S.A.A. and D.A.E.). If there was disagreement about the semiological analyses, a third senior investigator (N.A.K.) reviewed the video-EEG to resolve the dispute. The researchers designed a checklist containing all the items to be noted regarding the detailed semiological characteristics of the ictal events to be recorded by the physician (Supplementary material), including trunk/head/eye movements, ictal hyperventilation, verbalization, and responsiveness. Ictal emotional signs were verified, and the duration of each attack was recorded in seconds.

Clinical events were classified according to the functional seizure classification system described by Szabó et al. [17]: Minor motor: a localized motor accompanied by retained responsiveness; Major motor: complex movements involving several limbs, including hypermotor movements, body rigidity, pelvic thrusting with impaired consciousness; Dialeptic: unresponsiveness attacks without motor features; Nonepileptic auras: subjective sensations without any external manifestations, Mixed type: combinations of the above seizure types.

### Video-EEG protocol

The Video-EEG study was performed using a Nihon Kohden Neurofax EEG-1200 apparatus (Tokyo, Japan). Electrode placement was done according to the international 10–20 system with additional electrodes at T1/T2 positions and one channel for ECG recording.

In addition to hyperventilation and photic stimulation, which are used conventionally in EEG recordings, verbal suggestion techniques were occasionally used to explain to the patient that recording the habitual event would be necessary to guide treatment. The earlier the event occurs, the sooner the study could be discontinued, allowing the patient to go home. Patient-specific triggers were also used whenever possible, e.g., if events were triggered by sleep deprivation or excessive concentration, patients were instructed to avoid sleep or bring their study books.

The patient or their accompanying person was given the event button and instructed to press it whenever they were aware of the event of interest. The recording technician also marked abnormal movement or behavior, performed awareness testing during the event, and asked about the post-event amnesia. The technician confirmed with the patient or their accompanying person if the event recorded was that of interest. Standard safety procedures for PWE in the epilepsy monitoring unit were also taken into consideration to avoid any morbidity.

### Ethical statement

The study was made in agreement with the Declaration of Helsinki. The ethical approval was achieved by the Research ethical committee of Cairo University (N-217–2023).

### Statistical analysis

Microsoft Excel 2016 was used for data entry, and the statistical package for social science (SPSS, version 24) was used for data analysis. Data were explored for normal or skewed distribution using the Kolmogorov–Smirnov/Shapiro–Wilk’s test. Simple descriptive statistics in arithmetic mean and standard deviation were used to summarize numerical variables, while frequencies and percentages were used for categorical ones. The bivariate relationship was displayed in cross-tabulations, and proportions were compared using the chi-square and Fisher’s exact tests where appropriate. The *P-*value was calculated to assess statistical significance with values less than 0.05 considered statistically significant.

## Results

This study included 54 children and 65 adults diagnosed with functional seizures. The median age of the pediatric group was 13 years (IQR 10–15), while the median age of the adult group was 26.5 years ( IQR 22–35). Table 1 summarizes the detailed clinical characteristics of both groups.

**Table 1:** Clinical characteristics of pediatric and adult groups with PNES

		Pediatric PNES *n* (%)	Adult PNES *n* (%)	*P*-value
Sex	Female	28 (51.9%)	37 (56.9%)	0.580
	Male	26 (48.1%)	28 (43.1%)	
Family history of epilepsy	No	42 (77.8%)	55 (84.6%)	0.339
	Yes	12 (22.2%)	10 (15.4%)	
Family history of psychiatric illness	No	52 (96.3%)	61 (93.8%)	0.688
	Yes	2 (3.7%)	4 (6.2%)	

Twenty-three patients had concurrent epilepsy and functional seizures (13 in the pediatric group and 10 in the adult group). Of those with comorbid epilepsy (*n* = 23), 15 had focal epilepsy (5 with temporal lobe epilepsy [TLE] and 10 with extratemporal lobe epilepsy), while eight had generalized epilepsy. Twenty-eight others reported a history of taking ASMs prior to an incorrect epilepsy diagnosis.

Regarding semiological categorization, minor motor seizures were the most common semiology among adult and pediatric groups. However, the frequency of major motor seizures was significantly higher in adults than in pediatrics (38.5% vs. 16.7%). On the other hand, the pediatric group had significantly more minor motor events than adults (61.1% vs. 40.0%). Other semiological categorizations showed no significant differences between the two groups (Table 2) (Video S1 in Supplementary Information).

**Table 2:** Semiological categorization in pediatric PNES versus adult PNES

		Pediatric PNES *n* (%)	Adult PNES *n* (%)	*P*-value
Major motor PNES	Yes	9 (16.7%)	25 (38.5%)	0.009
	No	45 (83.3%)	40 (61.5%)	
Minor motor PNES	Yes	33 (61.1%)	26 (40.0%)	0.022
	No	21 (38.9%)	39 (60.0%)	
Dialeptic PNES	Yes	11 (20.4%)	7 (10.8%)	0.146
	No	43 (79.6%)	58 (89.2%)	
Nonepileptic auras	Yes	14 (25.9%)	16 (24.6%)	0.870
	No	40 (74.1%)	49 (75.4%)	
Mixed PNES	Yes	3 (5.6%)	1 (1.5%)	0.328
	No	51 (94.4%)	64 (98.5%)	

Most functional seizures in both groups were characterized by abrupt onset and absence of post-ictal manifestations. The general characteristics of functional seizures in both groups are summarized in Table 3, with no statistically significant difference between groups for any characteristic.

**Table 3:** General characteristics of PNES in pediatric versus adult group

		Pediatric PNES *n* (%)	Adult PNES *n* (%)	*P*-value
Frequency of attacks/month		6.5 (4–30)	8 (4–30)	0.368
Duration of attack (sec.)		60 (30–232.5)	120 (57.5–33)	0.123
Onset	Gradual	23 (42.6%)	27 (41.5%)	0.908
	Abrupt	31 (57.4%)	38 (58.5%)	
Stereotypical pattern	No	18 (33.3%)	31 (47.7%)	0.113
	Yes	36 (66.7%)	34 (52.3%)	
Occurrence alone	No	50 (92.6%)	58 (89.2%)	0.752
	Yes	4 (7.4%)	7 (10.8%)	
Responsiveness	Yes	28 (51.9%)	29 (44.6%)	0.431
	No	26 (48.1%)	36 (55.4%)	
Post-ictal	No	47 (87.0%)	61 (93.8%)	0.402
	Sleep	1 (1.9%)	0 (0.0%)	
	Fatigue	3 (5.6%)	1 (1.5%)	
	headache	2 (3.7%)	3 (4.6%)	
	Sensory	1 (1.9%)	0 (0.0%)	

Detailed semiological analysis revealed that pelvic thrusting was documented in 2 pediatric patients (3.7%) versus 6 adult patients (9.2%), back arching in 4 pediatric patients (7.4%) versus 3 adult patients (4.6%), and clenched fists in 5 pediatric patients (9.3%) versus 10 adult patients (15.4%), with no statistically significant difference between groups for any feature (Table 4).

**Table 4:** Detailed semiological patterns of PNES in pediatric versus adult groups

		Pediatric PNES *n* (%)	Adult PNES *n* (%)	*P*-value
Pelvic thrusting	Yes	2 (3.7%)	6 (9.2%)	0.290
	No	52 (96.3%)	59 (90.8%)	
Back arching	Yes	4 (7.4%)	3 (4.6%)	0.700
	No	50 (92.6%)	62 (95.4%)	
Side-to-side head movement	Yes	12 (22.2%)	22 (33.8%)	0.162
	No	42 (77.8%)	43 (66.2%)	
Hyper-extended neck	Yes	0 (0.0%)	2 (3.1%)	0.500
	No	54 (100.0%)	63 (96.9%)	
Teeth clenching	Yes	1 (1.9%)	6 (9.2%)	0.125
	No	53 (98.1%)	59 (90.8%)	
Tip tongue biting	Yes	2 (3.7%)	9 (13.8%)	0.109
	No	52 (96.3%)	56 (86.2%)	
Clenched fists	Yes	5 (9.3%)	10 (15.4%)	0.316
	No	49 (90.7%)	55 (84.6%)	
Ictal eye closure	Yes	17 (31.5%)	29 (44.6%)	0.143
	No	37 (68.5%)	36 (55.4%)	
Ictal blinking	Yes	3 (5.6%)	3 (4.6%)	1.000
	No	51 (94.4%)	62 (95.4%)	
Ictal eye deviation	Yes	2 (3.7%)	2 (3.1%)	1.000
	No	52 (96.3%)	63 (96.9%)	
Ictal eye-rolling	Yes	1 (1.9%)	1 (1.5%)	1.000
	No	53 (98.1%)	64 (98.5%)	
Ictal crying	Yes	4 (7.4%)	3 (4.6%)	0.700
	No	50 (92.6%)	62 (95.4%)	
Ictal fear	Yes	3 (5.6%)	2 (3.1%)	0.658
	No	51 (94.4%)	63 (96.9%)	
Ictal pain	Yes	7 (13.0%)	12 (18.5%)	0.415
	No	47 (87.0%)	53 (81.5%)	
Ictal laughter	Yes	1 (1.9%)	0 (0.0%)	0.454
	No	53 (98.1%)	65 (100.0%)	
Ictal hyperventilation	Yes	9 (16.7%)	4 (6.2%)	0.067
	No	45 (83.3%)	61 (93.8%)	
Ictal Verbalization	No	45 (83.3%)	58 (89.2%)	0.225
	Verbal	1 (1.9%)	3 (4.6%)	
	Non-verbal	8 (14.8%)	4 (6.2%)	

Ictal pain was described by 7 pediatrics (13.0%) versus 12 adults (18.5%), ictal fear by 3 pediatrics (5.6%) versus 2 adults (3.1%), while ictal crying was noticed in 4 pediatrics (7.4%) versus 3 adults (4.6%), with no statistically significant difference regarding any of them. Detailed semiological patterns of functional seizures in pediatric versus adult groups are illustrated in Table 4.

## Discussion

The present study established that the clinical semiology of functional seizures varies between pediatric and adult populations. While minor motor events are the most common functional seizures in both age groups, they occur more frequently in pediatric subjects. Conversely, adults are more likely to present with major motor functional seizures. The difference in semiology emphasizes the need for age-specific diagnostic and management approaches.

Studies illustrating semiological differences in functional seizures between pediatrics (< 18 years old) and adults (> 18 years old) are limited. Notably, the heterogeneity among studies may be attributed to variability in methodology; different populations studied, inter-observer variation, coexistent epilepsy, and available semiological classifications.

A large multi-center study described notable differences in the semiology of functional seizures across the lifespan [18]. In particular, the complexity of motor components of functional seizures increases with age. This is in line with the current results, as the frequency of major motor seizures was significantly higher in adults than in the pediatric group. Conversely, the pediatric group experienced a significantly higher frequency of minor motor seizures than the adult group.

Similar results were found when functional seizures were categorized by Alessi et al. [19] into four major groups according to Griffith’s classification [20]: major motor, minor motor, catatonic events, and subjective (aura). Children mostly displayed minor motor events in functional seizures, as opposed to the major motor events, which were more typical in adults.

Another study investigated functional seizures, focusing on the relationship between the age of onset and clinical semiology, and grouped them into two categories: juvenile-onset (< 18 years) and adult-onset (≥ 18 years). Clinical manifestations were similar in both groups, apart from ictal eye closure being more prevalent in juvenile-onset functional seizures [21].

Possible explanations for semiological differences between the two age groups are that motor events are difficult to execute and may rely on a history of witnessing an event [22]. The different psychopathology underlying functional seizures in pediatrics compared to adults could be another potential explanation [23, 24]. Younger subjects adopt different coping strategies to traumatic events and stressors than older subjects, in addition to the increasing prevalence of chronic medical conditions and stressors that increase with age [25].

The semiology might also be influenced by cultural differences. In a previous cross-cultural study of pediatric functional seizure, compared to other regions, Iranian patients exhibited more prevalent generalized motor seizures and ictal injuries, while unresponsiveness, though observed across all regions, was more prevalent in the middle eastern Iranian subjects than in western subjects [26].

Notably, no single symptom or sign could differentiate the pediatric and adult groups in the current study. Hence, it was important to establish classifications of functional seizures based on the predominant symptoms [27].

Most events were abrupt in onset, which is consistent with previous studies [15, 20]. Ictal eye closure was observed in 44.6% of adults and 31.5% of pediatric patients. Ictal eye closure has been reported to have a sensitivity of up to 58% and a specificity of 80% for diagnosing functional seizures [22].

Although psychosomatic symptoms change frequently within a patient, the semiology of functional seizure attacks is usually consistent in both the pediatric and adult populations [28]. The current study showed that most functional seizure events belonged to the same semiological type; 66.7% of pediatric patients and 52.3% of adult cases had stereotypical patterns.

Furthermore, although negative emotional signs like crying, moaning, and screaming have been considered important markers of pediatric functional seizures [29], none of them significantly differ between the adult and pediatric groups.

Likewise, pelvic thrusting is considered typical of adult functional seizures by [17]; however, it was observed in the present sample at 9.2% and 3.7% in adults and pediatrics, respectively, with no significant difference.

This study had some limitations that had to be acknowledged. First, some important data (e.g., psychiatric comorbidities, medications, parental education, financial status, etc.) were lacking, as the data collection relied on the available documentation. Future studies aiming to recognize the association between certain semiologies in functional seizures and underlying psychopathology are warranted. Also, we had to acknowledge the challenge of interpreting the non-epileptic auras in children. Additionally, the small sample size of patients with concurrent epilepsy and functional seizures precluded further analysis to detect differences in semiology between those patients and others without epilepsy.

## Conclusions

There were more similarities than differences in the functional seizure semiology between pediatric and adult patients. The minor motor type was the most common semiology in both age groups, yet it was more prevalent in pediatric patients than adult patients. In contrast, major motor events were more characteristic of the adult group. A detailed evaluation of underlying psycho-social stressors concerning presenting semiology is highly encouraged.

## Supplementary Information

Supplementary Material 1.

Supplementary Material 2.

## Data Availability

Authors report that the datasets used and/or analyzed during the current study are available from the corresponding author upon reasonable request.

## Abbreviations

ASM: Anti-seizure Medications
CUEU: Cairo University Epilepsy Unit
ECG: Electrocardiogram
EEG: Electroencephalogram
EMU: Epilepsy Monitoring Units
FND: Functional Neurological Disorder
IQR: Interquartile Range
PWE: Patients with Epilepsy
TLE: Temporal Lobe Epilepsy
