# Supplementary material for: Semiological differences of functional seizures between pediatrics and adults: video electroencephalography analysis
Source: Acta Epileptol. 2026 Feb 2;8:3. doi: 10.1186/s42494-025-00236-0 (PMC12862905; doi:10.1186/s42494-025-00236-0)
Supplement: Supplementary file 1 — Supplementary Material 1. [file 42494_2025_236_MOESM1_ESM.docx]

| Duration of attack (sec.) | | |
| --- | --- | --- |
| Onset | Gradual | |
|  | Abrupt | |
| Ictal | Responsiveness |  |
|  | Pelvic thrusting |  |
|  | Back arching |  |
|  | Side -to-side head movement |  |
|  | Hyper-extended neck |  |
|  | Teeth clenching |  |
|  | Tip tongue biting |  |
|  | Clenched fists |  |
|  | Ictal eye closure |  |
|  | Ictal blinking |  |
|  | Ictal eye deviation |  |
|  | Ictal eye-rolling |  |
|  | Ictal crying |  |
|  | Ictal fear |  |
|  | Ictal pain |  |
|  | Ictal laughter |  |
|  | Ictal hyperventilation |  |
|  | Ictal Verbalization |  |
|  | Urinary incontinence |  |
